# Supplementary material for: Myasthenia Gravis Activities of Daily Living Deep Phenotyping and Longitudinal
Source: medRxiv. 2026 Oct 28:2026.10.26.26366495. Preprint. [Version 1] doi: 10.64898/2026.10.26.26366495 (PMC13734193; doi:10.64898/2026.10.26.26366495)
Supplement: Supplementary file 1 [file NIHPP2026.10.26.26366495v1-supplement-1.pdf]

medRxiv preprint doi: https://doi.org/10.64898/2026.10.26.26366495; this version posted October 28, 2026. The copyright holder for this preprint (which was not certified by peer review) is the author/funder, who has granted medRxiv a license to display the preprint in perpetuity.
It is made available under a CC-BY 4.0 International license .

# Supplementary Figures

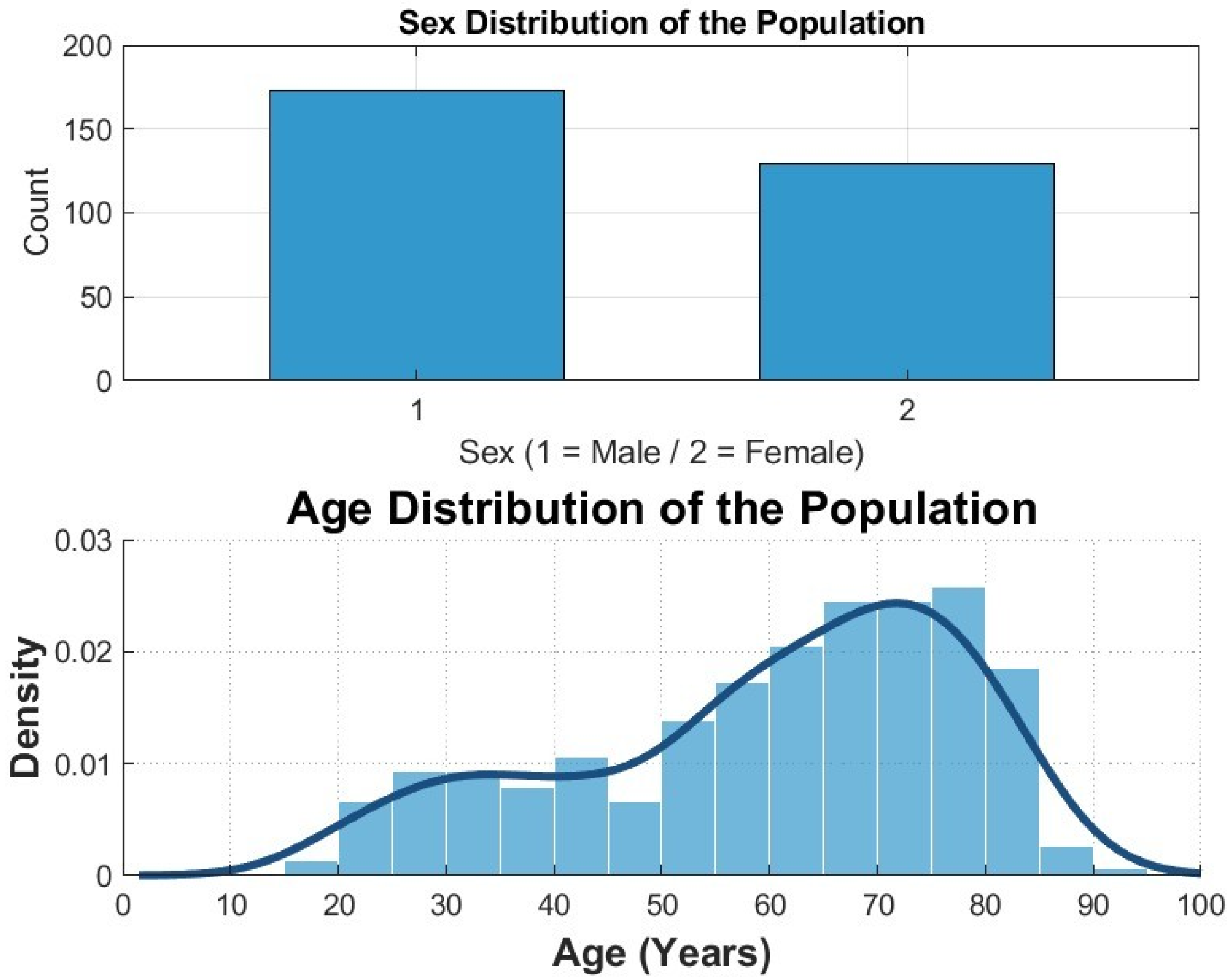


Figure A1

Explore Study

medRxiv preprint doi: https://doi.org/10.64898/2026.10.26.26366495; this version posted October 28, 2026. The copyright holder for this preprint (which was not certified by peer review) is the author/funder, who has granted medRxiv a license to display the preprint in perpetuity.
It is made available under a CC-BY 4.0 International license .

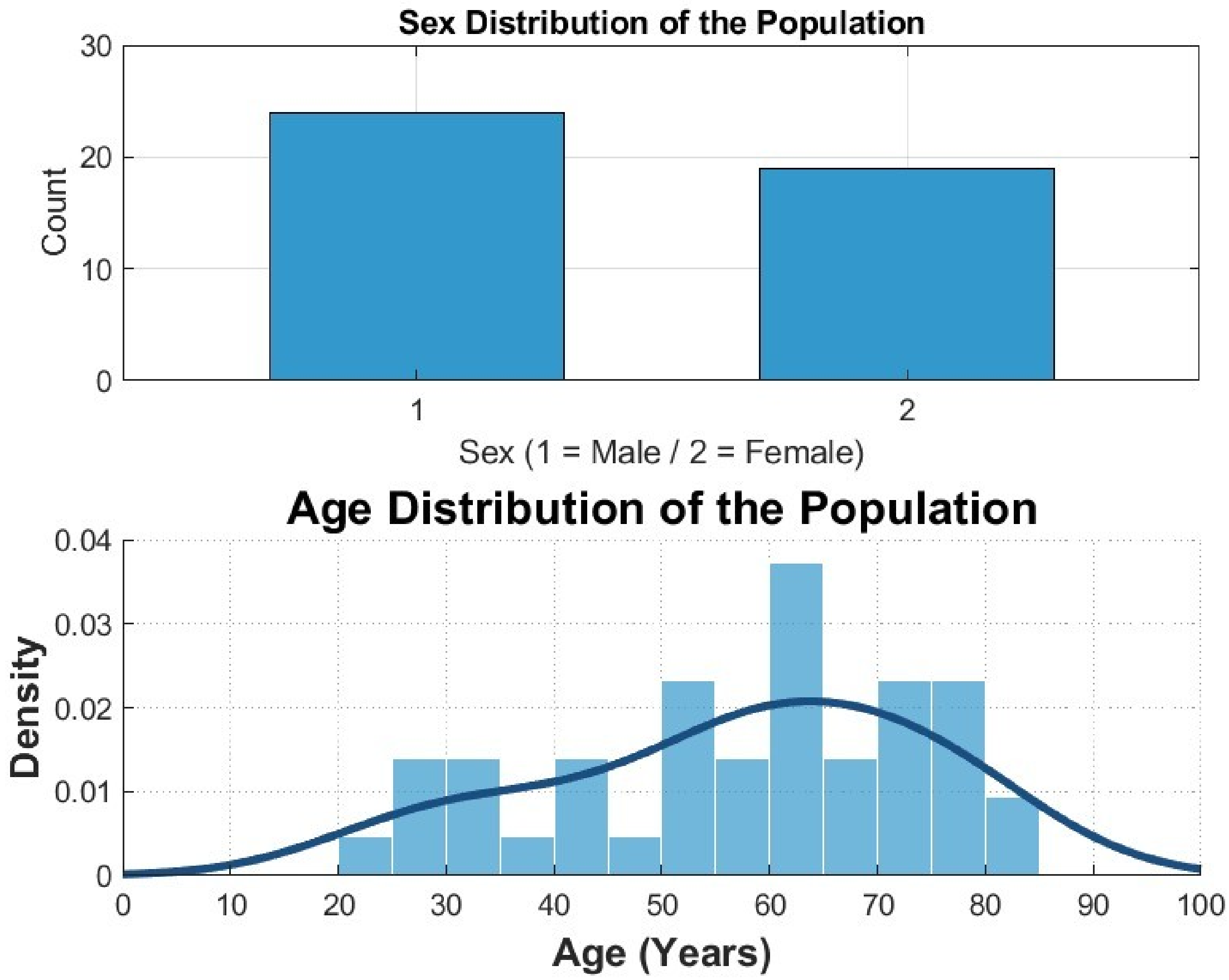


Figure A2

BeatMG Clinical Trial.

medRxiv preprint doi: https://doi.org/10.64898/2026.10.26.26366495; this version posted October 28, 2026. The copyright holder for this preprint (which was not certified by peer review) is the author/funder, who has granted medRxiv a license to display the preprint in perpetuity.
It is made available under a CC-BY 4.0 International license .

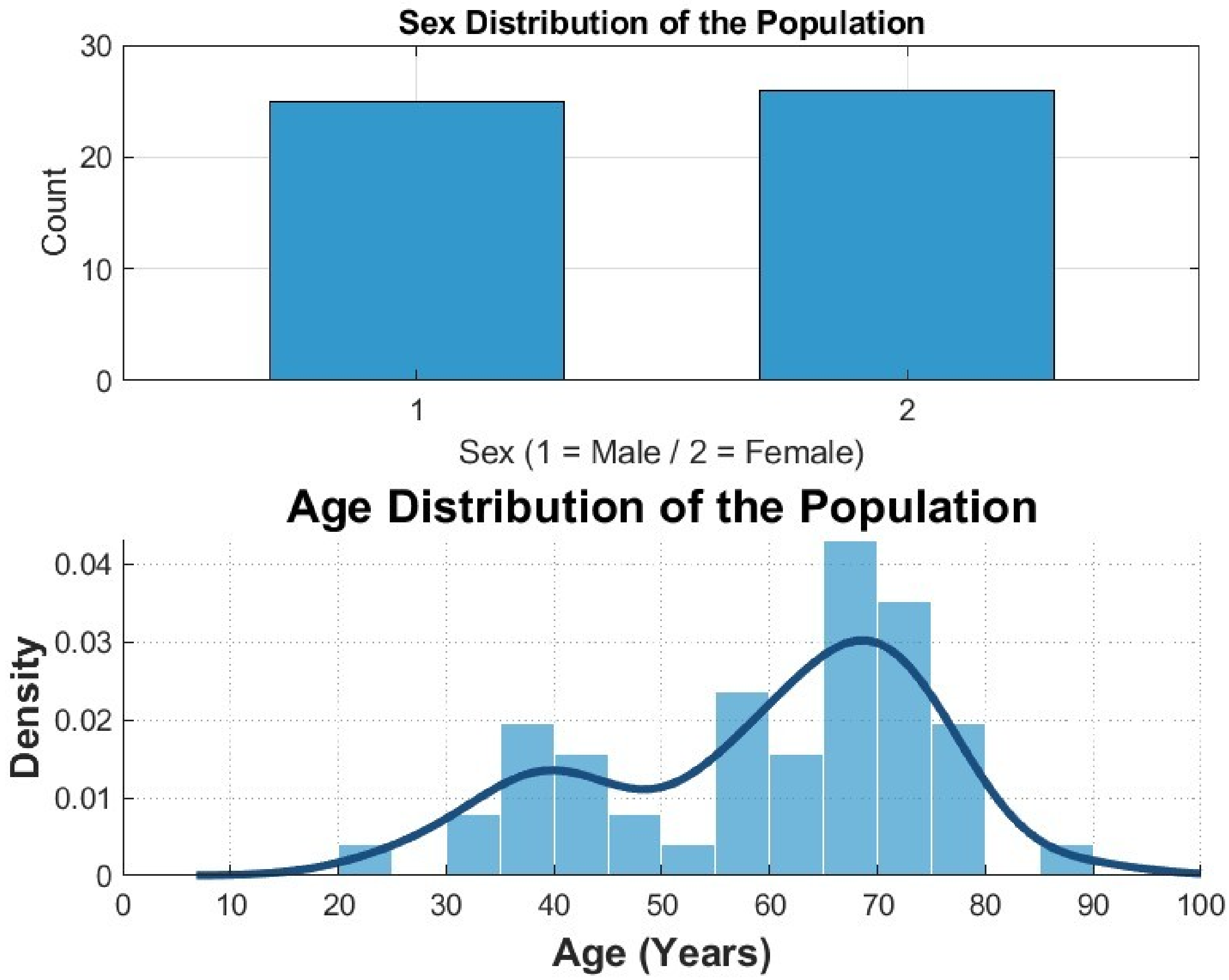


Figure A3

MGnet Telemedicine study

medRxiv preprint doi: https://doi.org/10.64898/2026.10.26.26366495; this version posted October 28, 2026. The copyright holder for this preprint
(which was not certified by peer review) is the author/funder, who has granted medRxiv a license to display the preprint in perpetuity.
It is made available under a CC-BY 4.0 International license .

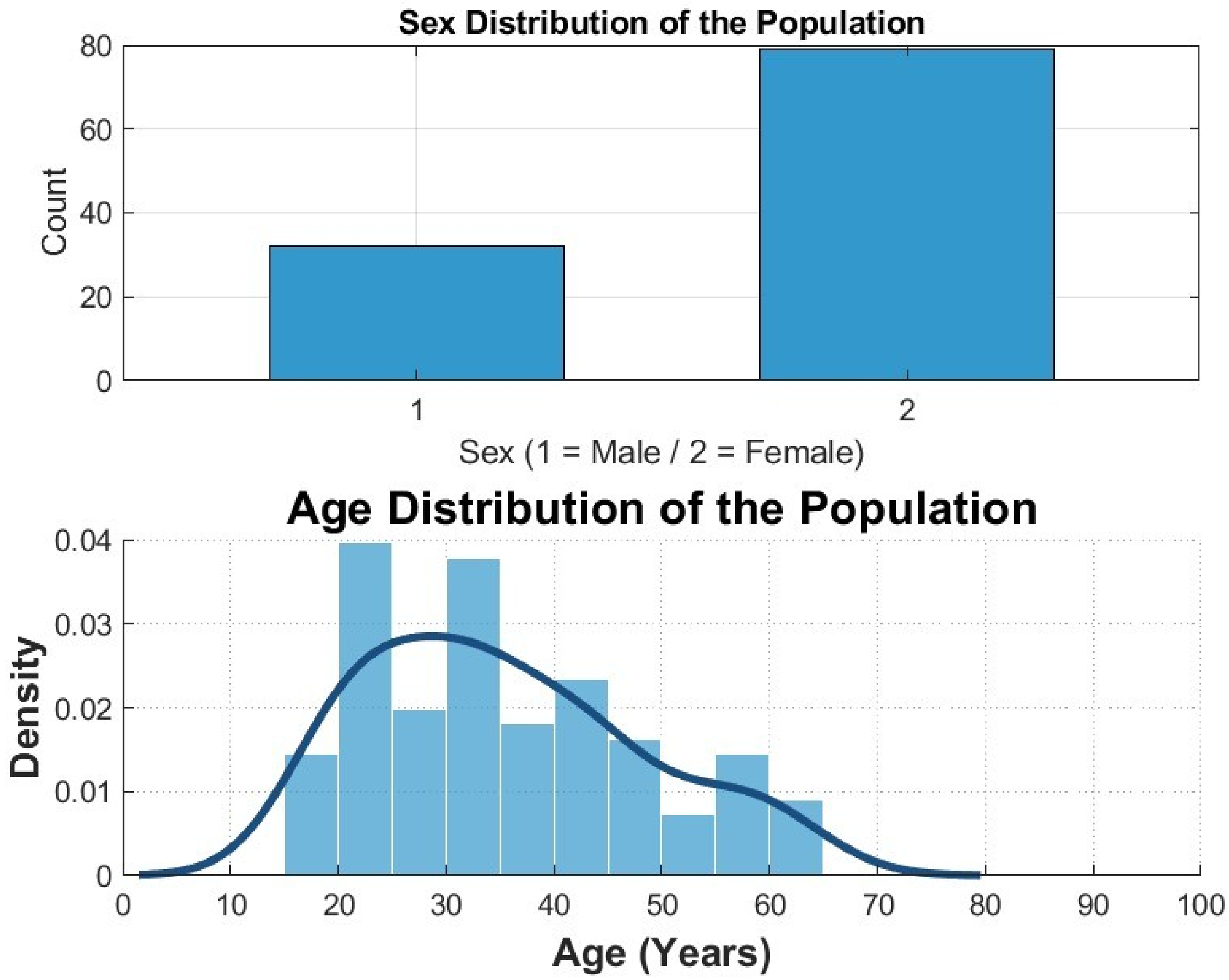


Figure A4

MGTX Clinical Trial

medRxiv preprint doi: https://doi.org/10.64898/2026.10.26.26366495; this version posted October 28, 2026. The copyright holder for this preprint (which was not certified by peer review) is the author/funder, who has granted medRxiv a license to display the preprint in perpetuity.
It is made available under a CC-BY 4.0 International license .

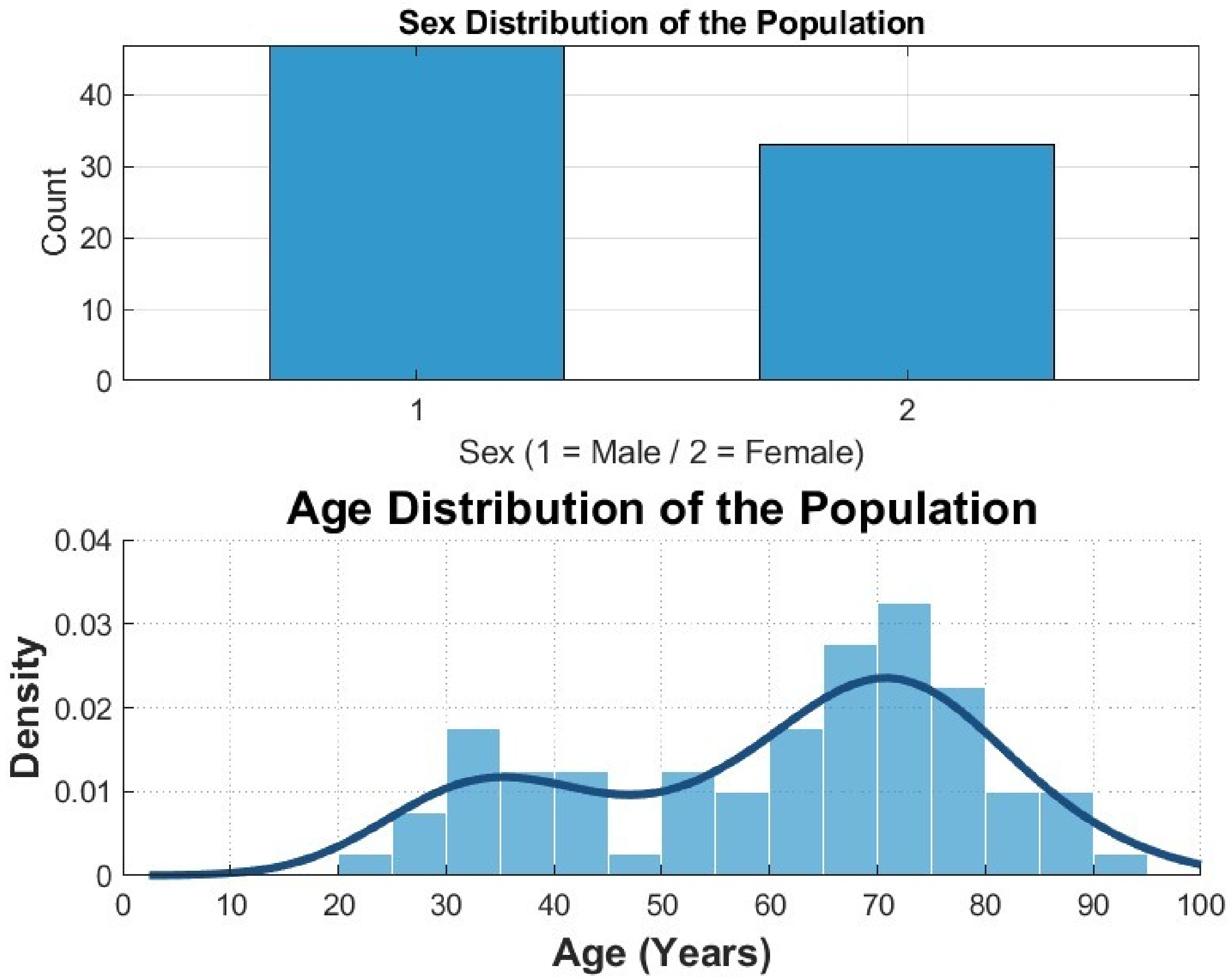


Figure A5

MSG Clinical Trial

medRxiv preprint doi: https://doi.org/10.64898/2026.10.26.26366495; this version posted October 28, 2026. The copyright holder for this preprint (which was not certified by peer review) is the author/funder, who has granted medRxiv a license to display the preprint in perpetuity.
It is made available under a CC-BY 4.0 International license .

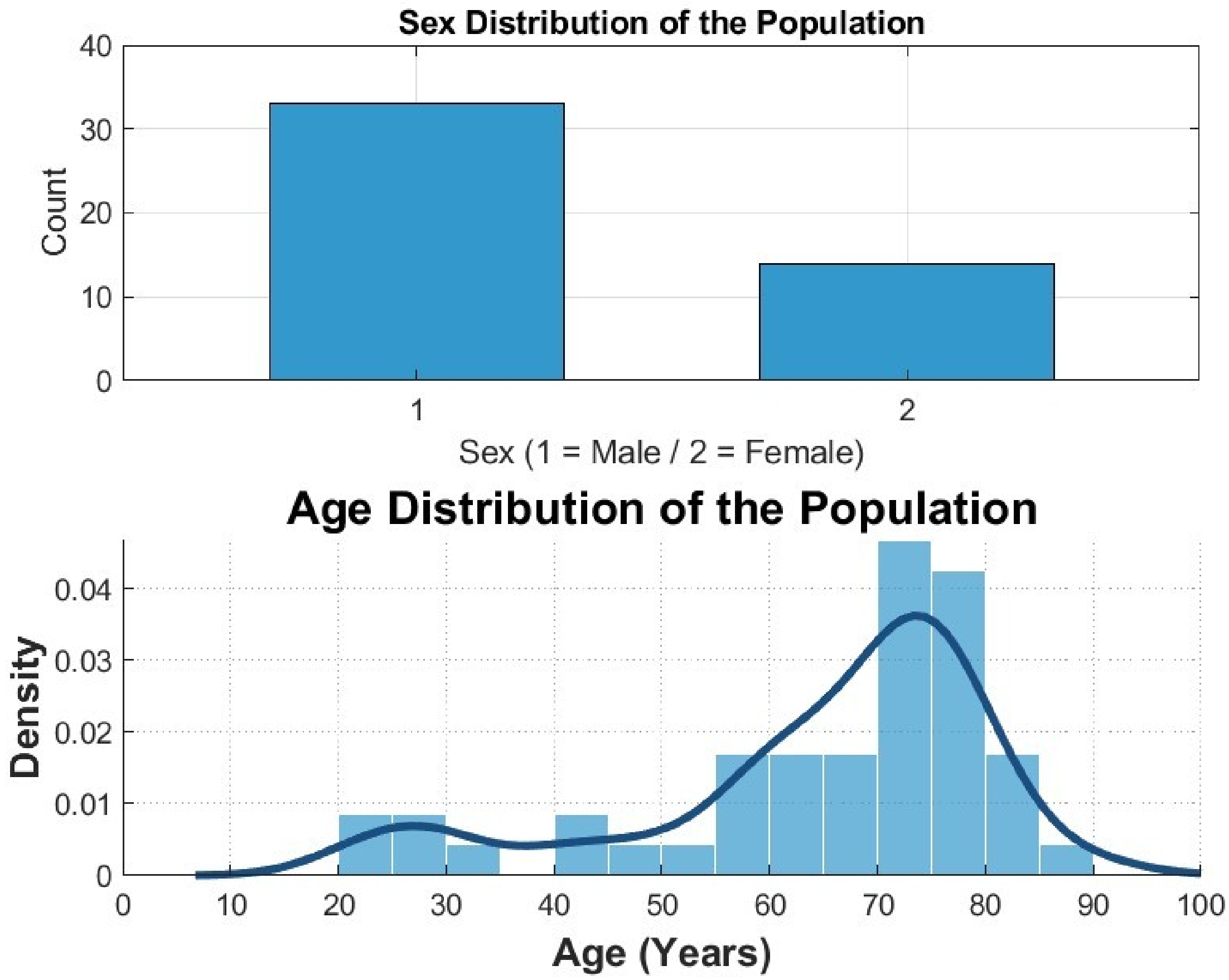


Figure A6

Rinomax Clinical Trial.

medRxiv preprint doi: https://doi.org/10.64898/2026.10.26.26366495; this version posted October 28, 2026. The copyright holder for this preprint (which was not certified by peer review) is the author/funder, who has granted medRxiv a license to display the preprint in perpetuity.
It is made available under a CC-BY 4.0 International license .

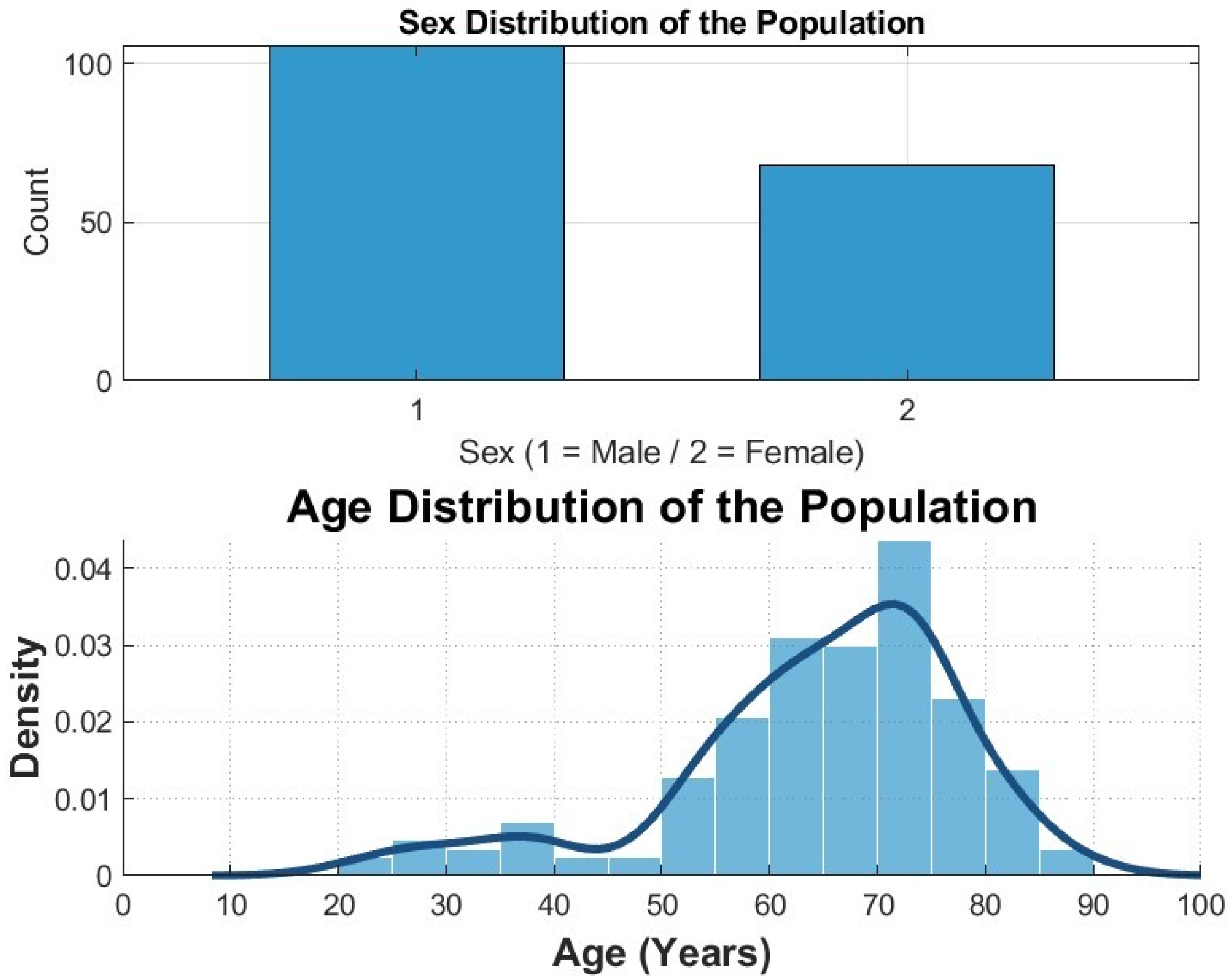


Figure A7

Promise Clinical Trial.

medRxiv preprint doi: https://doi.org/10.64898/2026.10.26.26366495; this version posted October 28, 2026. The copyright holder for this preprint (which was not certified by peer review) is the author/funder, who has granted medRxiv a license to display the preprint in perpetuity.
It is made available under a CC-BY 4.0 International license .

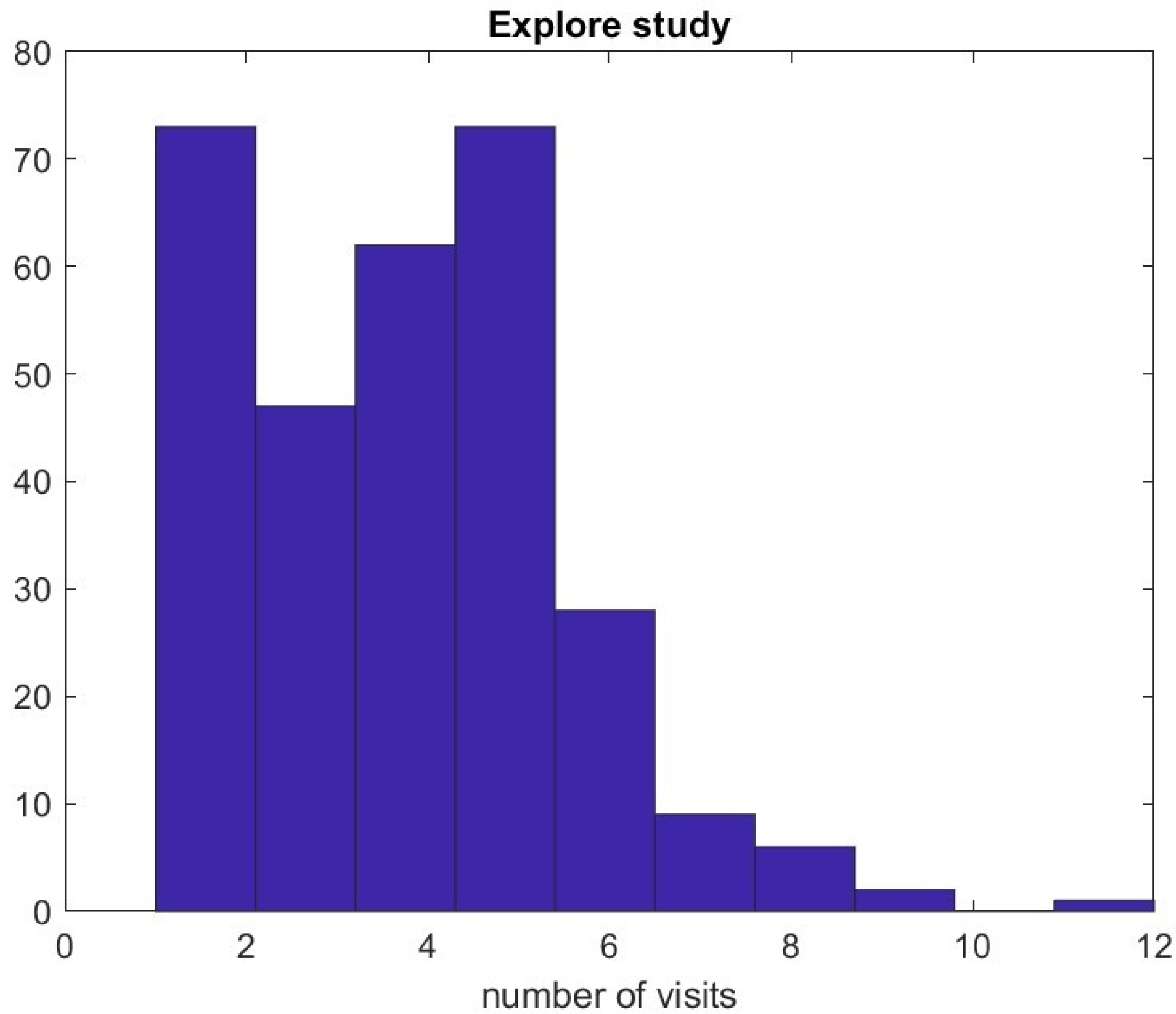


Figure A8

Distribution of the number of visits per patient in Explore.

medRxiv preprint doi: https://doi.org/10.64898/2026.10.26.26366495; this version posted October 28, 2026. The copyright holder for this preprint (which was not certified by peer review) is the author/funder, who has granted medRxiv a license to display the preprint in perpetuity.
It is made available under a CC-BY 4.0 International license .

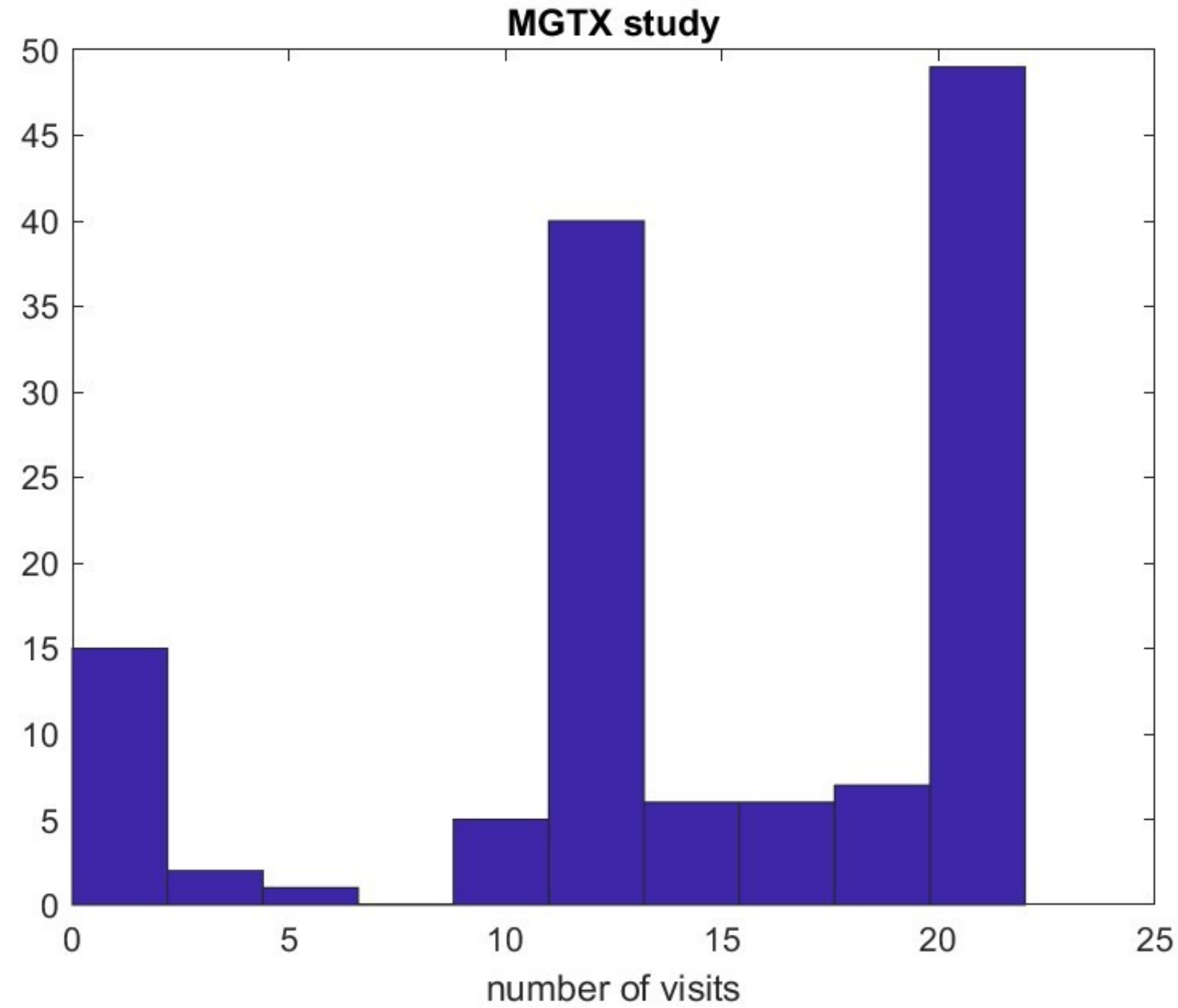


Figure A 9

Distribution of the number of visits per patient in MGTX.

MGADL COMBINED MACRO & MICRO OUTLIER REPORT

==================================================================================
=

| Study | Valid/Total N | Mean Total (SD) | Median | Highest Domain Incidence (>0) |
|---|---|---|---|---|
| beatMG | 705 / 728 | 3.39 (3.13) | 3.0 | Vision (48.2%) |
| MGTX | 1907 / 1908 | 2.40 (2.92) | 1.0 | Eyelid drop (36.8%) |
| MGnet | 104 / 104 | 3.66 (3.62) | 2.0 | Vision (41.3%) |
| Explore | 1176 / 1176 | 2.85 (3.22) | 2.0 | Eyelid drop (40.6%) |
| MSG | 551 / 553 | 3.54 (3.25) | 3.0 | Eyelid drop (49.9%) |
| Rinomax | 328 / 328 | 3.96 (3.38) | 3.0 | Breath (55.8%) |

medRxiv preprint doi: https://doi.org/10.64898/2026.10.26.26366495; this version posted October 28, 2026. The copyright holder for this preprint (which was not certified by peer review) is the author/funder, who has granted medRxiv a license to display the preprint in perpetuity.
It is made available under a CC-BY 4.0 International license .

| | | | | |
|---|---|---|---|---|
| Promise | 488 / 1092 | 4.26 (3.15) | 4.0 | Vision (63.3%) |

Table A10

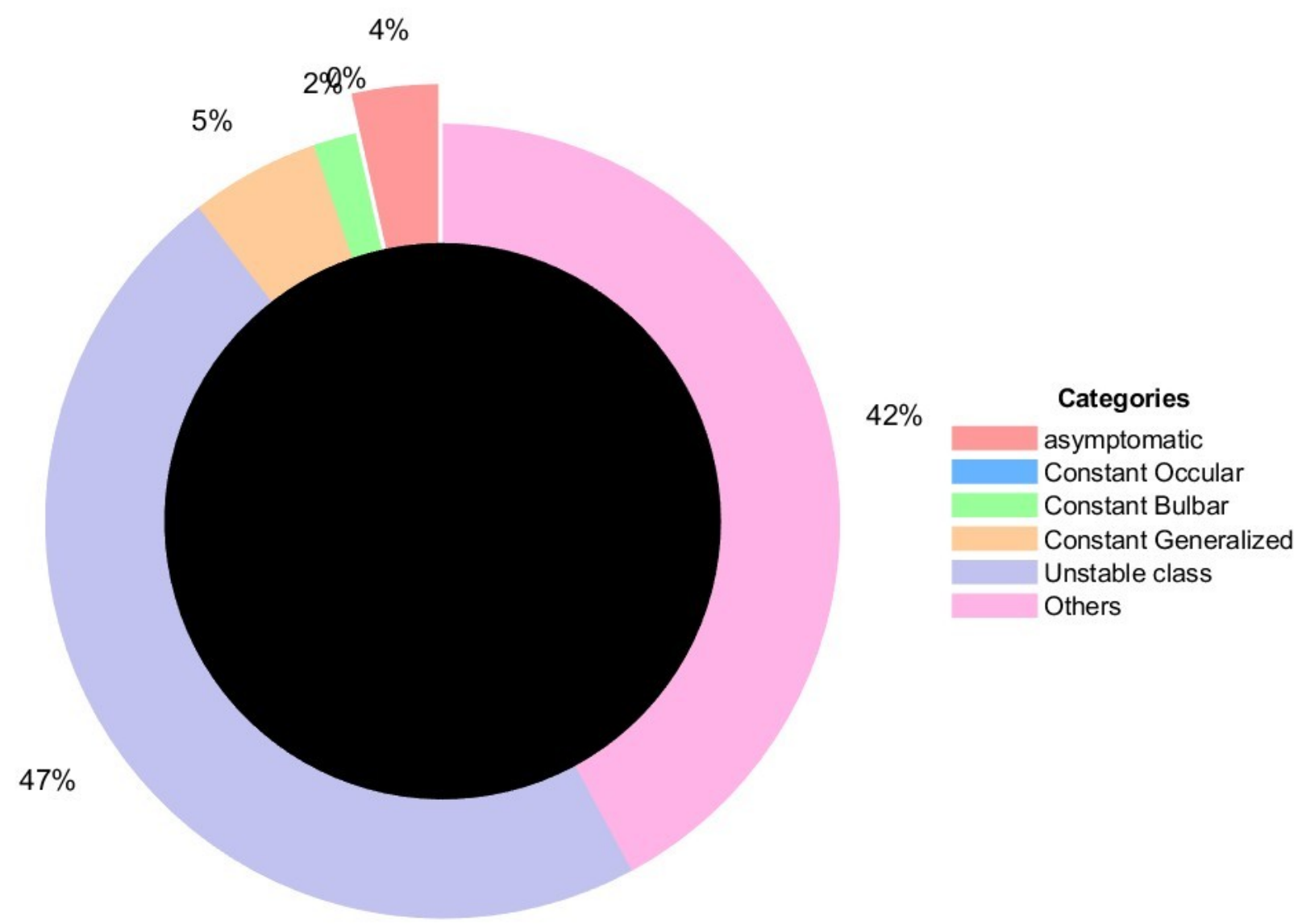

medRxiv preprint doi: https://doi.org/10.64898/2026.10.26.26366495; this version posted October 28, 2026. The copyright holder for this preprint (which was not certified by peer review) is the author/funder, who has granted medRxiv a license to display the preprint in perpetuity.
It is made available under a CC-BY 4.0 International license .

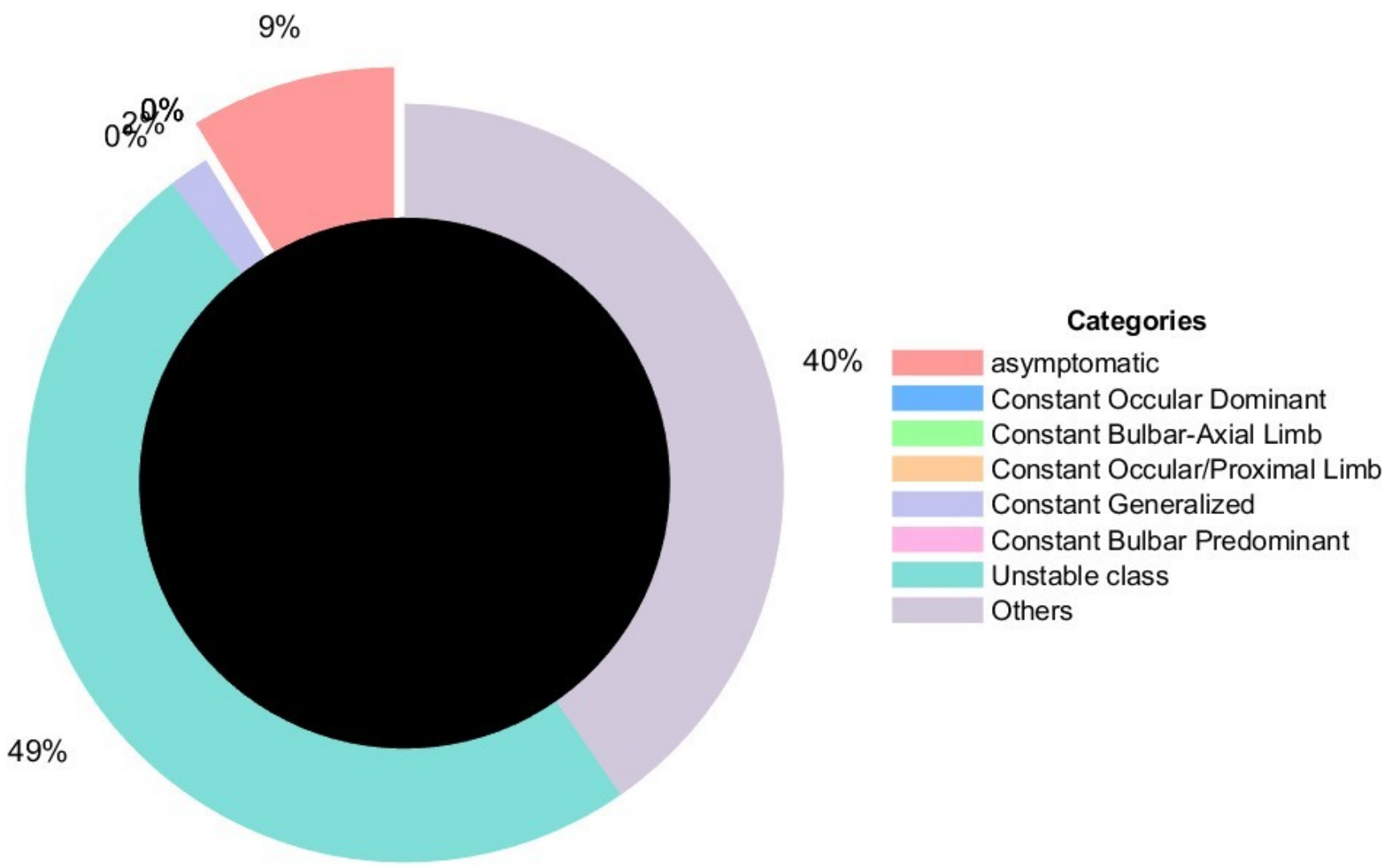


Figure A11

Stability of patient phenotypes in PROMISE, using the three-cluster model (top) and the five-cluster model (bottom).

Approximately 47%–49% of patients had unstable longitudinal MG-ADL phenotypes, defined as a change in cluster assignment at least once over three visits. The analysis was restricted to patients with at least four visits, which represented the large majority of the cohort. Instability was more frequent than in MGTX and Explore.

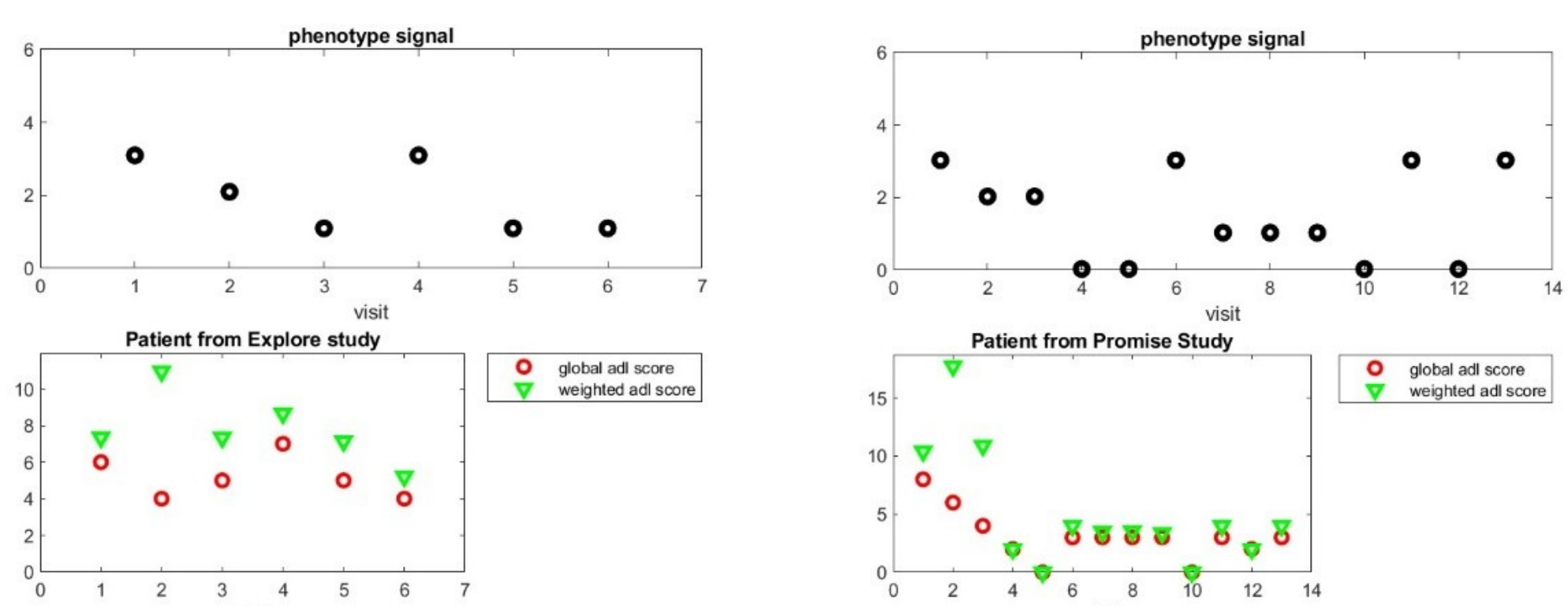

medRxiv preprint doi: https://doi.org/10.64898/2026.10.26.26366495; this version posted October 28, 2026. The copyright holder for this preprint (which was not certified by peer review) is the author/funder, who has granted medRxiv a license to display the preprint in perpetuity.
It is made available under a CC-BY 4.0 International license .

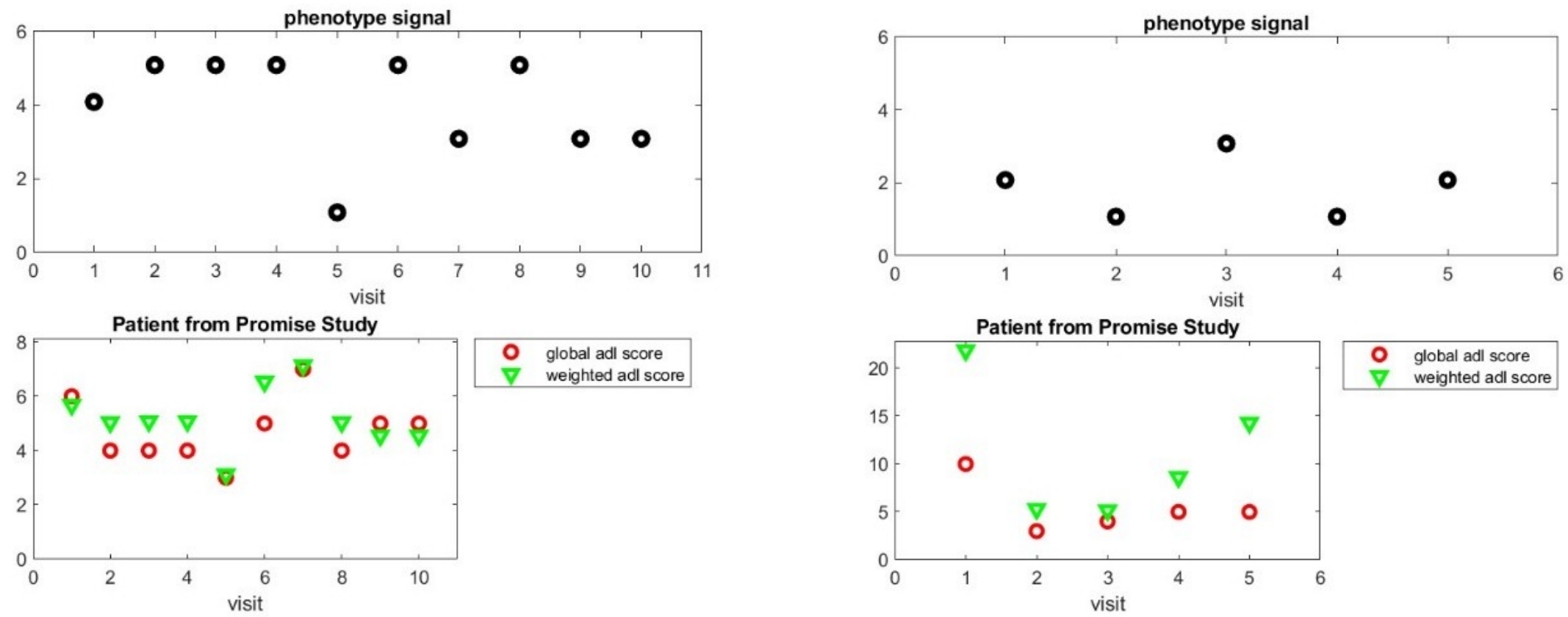


Figure A12

Selected trajectories illustrate repeated transitions between phenotypes. In each example, changes in the aggregate score over time can differ from changes in the weighted score. By design, the weighted score is more sensitive to redistribution among individual items, because small item-level changes may have a substantial effect even when the aggregate score changes little.

medRxiv preprint doi: https://doi.org/10.64898/2026.10.26.26366495; this version posted October 28, 2026. The copyright holder for this preprint
(which was not certified by peer review) is the author/funder, who has granted medRxiv a license to display the preprint in perpetuity.
It is made available under a CC-BY 4.0 International license .

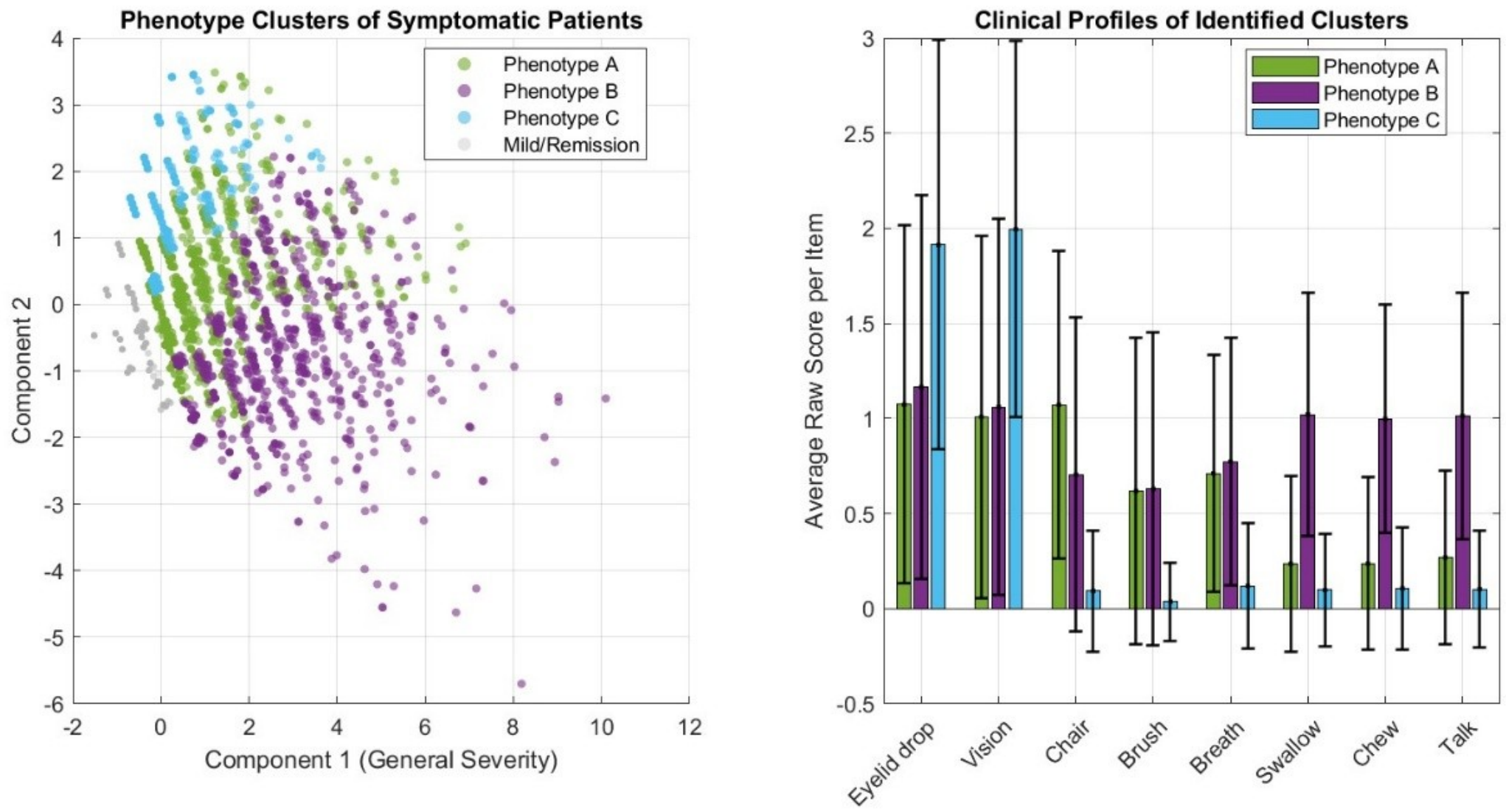


Figure A13

Classification using three clusters. The clusters corresponded broadly to ocular-dominant, bulbar-dominant, and generalized MG. This model did not separate the contributions of other items identified by PCA, including chair, brush, and breathing.

medRxiv preprint doi: https://doi.org/10.64898/2026.10.26.26366495; this version posted October 28, 2026. The copyright holder for this preprint (which was not certified by peer review) is the author/funder, who has granted medRxiv a license to display the preprint in perpetuity.
It is made available under a CC-BY 4.0 International license .

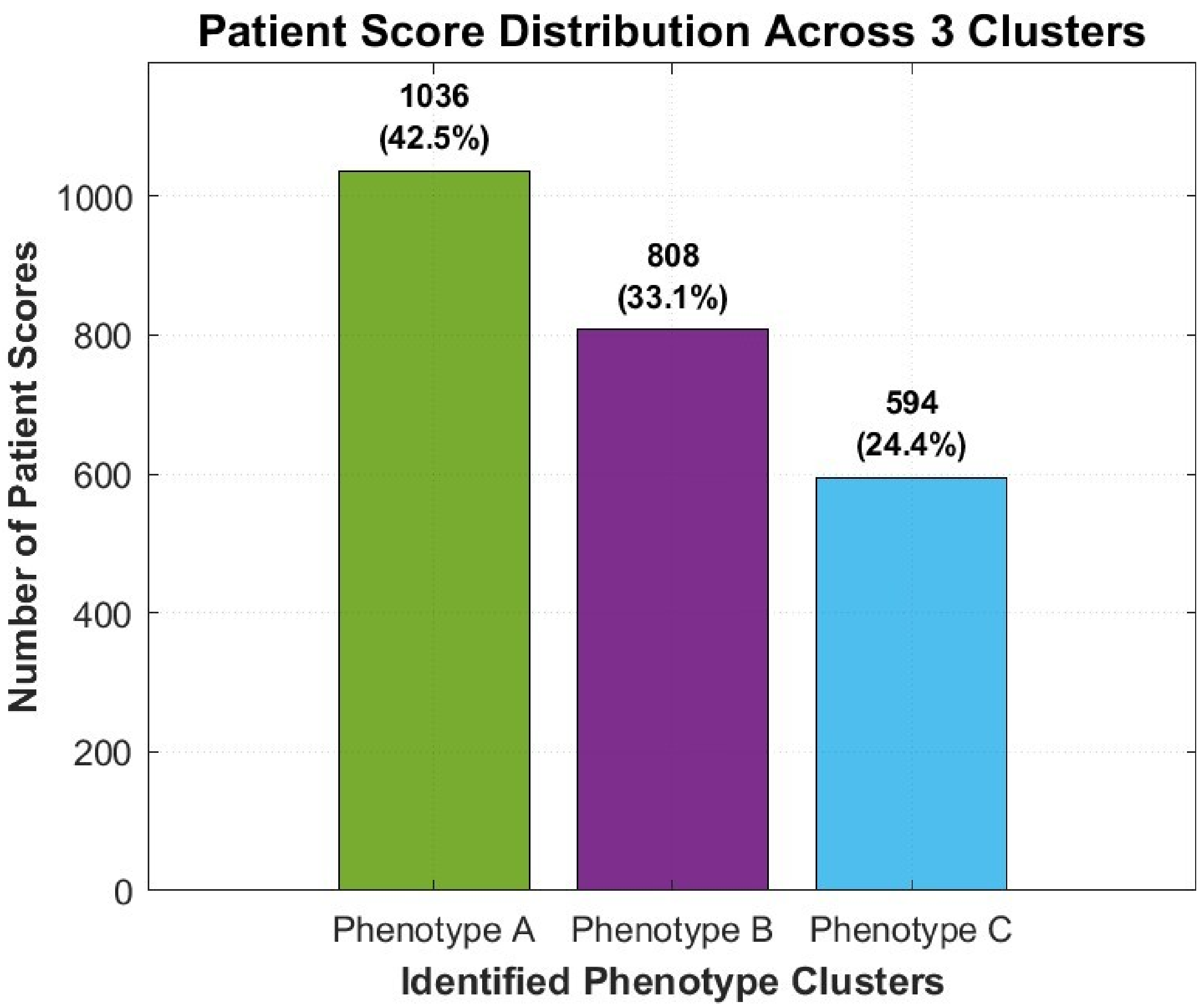


Figure A14

Distribution of symptomatic assessments across the three clusters.
